# Diet modulates brain network stability, a biomarker for brain aging, in young adults

**DOI:** 10.1073/pnas.1913042117

**Published:** 2020-03-03

**Authors:** Lilianne R. Mujica-Parodi, Anar Amgalan, Syed Fahad Sultan, Botond Antal, Xiaofei Sun, Steven Skiena, Andrew Lithen, Noor Adra, Eva-Maria Ratai, Corey Weistuch, Sindhuja Tirumalai Govindarajan, Helmut H. Strey, Ken A. Dill, Steven M. Stufflebeam, Richard L. Veech, Kieran Clarke

**Affiliations:** ^a^Department of Biomedical Engineering, Stony Brook University, Stony Brook, NY 11794;; ^b^Laufer Center for Physical and Quantitative Biology, Stony Brook University, Stony Brook, NY 11794;; ^c^Department of Physics and Astronomy, Stony Brook University, Stony Brook, NY 11794;; ^d^Athinoula A. Martinos Center for Biomedical Imaging, Massachusetts General Hospital and Harvard Medical School, Charlestown, MA 02129;; ^e^Department of Computer Science, Stony Brook University, Stony Brook, NY 11794;; ^f^Department of Applied Mathematics and Statistics, Stony Brook University, Stony Brook, NY 11794;; ^g^Laboratory of Metabolic Control, NIH/National Institute on Alcohol Abuse and Alcoholism, Rockville, MD 20852;; ^h^Department of Physiology, Anatomy, and Genetics, University of Oxford, Oxford OX1 3PT, United Kingdom

**Keywords:** glucose, ketone, beta-hydroxybutyrate, neural, brain

## Abstract

Epidemiological studies suggest that insulin resistance accelerates progression of age-based cognitive impairment, which neuroimaging has linked to brain glucose hypometabolism. As cellular inputs, ketones increase Gibbs free energy change for ATP by 27% compared to glucose. Here we test whether dietary changes are capable of modulating sustained functional communication between brain regions (network stability) by changing their predominant dietary fuel from glucose to ketones. We first established network stability as a biomarker for brain aging using two large-scale (*n* = 292, ages 20 to 85 y; *n* = 636, ages 18 to 88 y) 3 T functional MRI (fMRI) datasets. To determine whether diet can influence brain network stability, we additionally scanned 42 adults, age < 50 y, using ultrahigh-field (7 T) ultrafast (802 ms) fMRI optimized for single-participant-level detection sensitivity. One cohort was scanned under standard diet, overnight fasting, and ketogenic diet conditions. To isolate the impact of fuel type, an independent overnight fasted cohort was scanned before and after administration of a calorie-matched glucose and exogenous ketone ester (d-β-hydroxybutyrate) bolus. Across the life span, brain network destabilization correlated with decreased brain activity and cognitive acuity. Effects emerged at 47 y, with the most rapid degeneration occurring at 60 y. Networks were destabilized by glucose and stabilized by ketones, irrespective of whether ketosis was achieved with a ketogenic diet or exogenous ketone ester. Together, our results suggest that brain network destabilization may reflect early signs of hypometabolism, associated with dementia. Dietary interventions resulting in ketone utilization increase available energy and thus may show potential in protecting the aging brain.

## Significance

To better understand how diet influences brain aging, we focus here on the presymptomatic period during which prevention may be most effective. Large-scale life span neuroimaging datasets show functional communication between brain regions destabilizes with age, typically starting in the late 40s, and that destabilization correlates with poorer cognition and accelerates with insulin resistance. Targeted experiments show that this biomarker for brain aging is reliably modulated with consumption of different fuel sources: Glucose decreases, and ketones increase the stability of brain networks. This effect replicated across both changes to total diet as well as fuel-specific calorie-matched bolus, producing changes in overall brain activity that suggest that network “switching” may reflect the brain’s adaptive response to conserve energy under resource constraint.

Because the human brain is only 2% of the body’s volume but consumes over 20% of its energy (1, 2), it is particularly vulnerable to changes in metabolism. Dietary increase in glycemic load over the past 100 y has led to a national epidemic of insulin resistance (type 2 diabetes [T2D]) (3, 4), which has been identified by several large-scale epidemiological studies as an early risk factor for later-life dementia (5). For example, a post hoc analysis of the UK Whitehall II cohort study (*n* = 5,653) reported that those with diabetes showed a 45% faster decline in memory, a 29% faster decline in reasoning, and a 24% faster decline in global cognitive score and that the risk of accelerated cognitive decline in middle-aged patients with T2D is dependent on both disease duration and glycemic control (6). Similar results were reported using cohorts obtained from Israel (*n* = 897) (7) and the United States (*n* = 4,135) (8), the latter of which found the relationship between T2D and cognitive dysfunction to be evident even in younger adults. This marked association has led some researchers to propose that dementia may be the brain’s manifestation of metabolic disease (9).

This association is all the more surprising because, until quite recently, the brain was assumed to make use of purely insulin-independent transport of glucose into cells (GLUT3), utilizing neither insulin nor insulin transport (GLUT4). However, there now is rapidly accumulating evidence that insulin is directly relevant to neurons, brain aging, and associated memory deficits. For example, an early breakthrough study with radioactive insulin staining found that, contrary to the assumption that neurons did not utilize insulin, the rat brain was, in fact, densely populated with insulin receptors in both the hippocampus and cortex (10). Positron emission tomography in humans has demonstrated reduced glucose uptake in insulin-resistant participants versus healthy controls (11), suggesting that even the earliest stages of T2D induce hypometabolism of neurons, as with other cells in the body and as per brain glucose hypometabolism commonly seen in dementia. Finally, infusing insulin, without increasing glucose, has been shown to increase memory for Alzheimer’s disease patients (12). These clinical studies suggest that deleterious cognitive effects of insulin resistance may result from metabolic stress, as neurons gradually lose access to glucose. If so, it may be possible to bypass insulin resistance to refeed neurons by exploiting ketone bodies as an alternative fuel.

Endogenous ketone bodies, including d-β-hydroxybutyrate, are primarily produced in the liver from long- and medium-chain free fatty acids released from adipose tissue during hypocaloric/fasting states or food when following a low-carbohydrate/moderate-protein/high-fat diet (13). In rats, neurological and cognitive effects of glucocorticoid-induced insulin resistance in the hippocampus were reversed by ketone bodies (d-β-hydroxybutyrate) and mannose but not by either glucose or fructose (14). Likewise, in humans there is evidence that even as older brains become hypometabolic to glucose, neural uptake of ketone bodies remains unaffected, even for the most severe glucose hypometabolism endemic to Alzheimer’s disease (15, 16). Finally, lifelong hypocalorically induced ketosis preserves synaptic plasticity (17) and cognition (18) in elderly animals [chronological age equivalent to ∼87 to 93 human years (19)].

Beyond the ability to short-circuit insulin resistance, however, ketone bodies have other metabolic advantages (20–24) that may confer neurobiological benefits even to younger healthy individuals not yet in a deficit (hypometabolic) state. Of those advantages, one of the most fundamental is that, as cellular inputs, β-hydroxybutyrate molecules increase Gibbs free energy change for ATP by 27% compared to glucose (24). While it is currently unknown how increasing available energy might impact a healthy brain, one consequence suggested by prior animal data is an increase in neurotransmitter production. Eight- to ten-month-old mice, the chronological equivalent of ∼27- to 33-y-old humans (19), showed increased synaptic efficiency, low-theta band oscillations, and learning consolidation during intermittent-fasting-induced ketosis (25). Mechanistically, this increase in synaptic efficiency was linked to increased expression of the *N*-methyl-d-aspartate (NMDA) receptor for glutamate.

Here we test two hypotheses. First, we investigate the time course of brain aging in humans to determine whether there is evidence for a long-term degenerative process that lays the foundation for neurometabolic stress—decades before cognitive effects become evident. This is clinically critical because it identifies a window of time during which neurodegenerative effects may still be reversible if we can increase neurons’ access to fuel. Second, to isolate the role of energy in modulating this variable, we hold age constant while testing the neurobiological impact of switching the primary fuel source of the human brain from glucose to ketone bodies. The above translational results showed that fasting increases NMDA-driven synaptic efficiency (25); neurotransmission, in turn, has been shown to drive change in cerebral blood flow (26) and thus functional communication between brain regions measured by blood oxygen level–dependent (BOLD) functional MRI (fMRI) resting-state connectivity (27). Therefore, we expected that ketone bodies might improve fMRI-derived measures of neurobiological functioning, even in healthy younger adults.

To test these hypotheses, we proceeded in two stages. First, using independent large-scale human fMRI datasets, sampling across the adult life span (ages 18 to 88), we established a whole-brain-scale biomarker (network stability, defined as the brain’s ability to sustain functional communication between its regions) that robustly associates with brain aging. Second, we conducted two targeted experiments in humans, optimized for detection sensitivity at the single-participant level, to test the impact of manipulating fuel type: glucose versus ketone bodies, using both diet and bolus, on that biomarker. Of note, while translational studies tend to employ long-term (“lifelong”) dietary modifications—equivalent to 20 to 30 y of human life span—for our targeted experiments we deliberately focused on rapid effects (after 1 wk of the ketogenic diet and half an hour for the d-βHb ketone ester). This was done for three reasons. First, it permitted a within-subject design, thereby rigorously controlling for genetic and environmental differences between subjects. Second, it narrowed down the number of potential biological mechanisms to those capable of acting over minutes or days, rather than months, years, or decades. Finally, we maximized clinical relevance by using dietary modifications that would be realistic to implement by most individuals in real-world environments.

## Methods

### Life Span Neuroimaging Datasets.

To identify network stability across the life span, we analyzed two large-scale open-source 3 T fMRI resting-state datasets: Max Planck Institute Leipzig Mind-Brain-Body (28) (Leipzig: ages 20 to 85, *n* = 292) and Cambridge Centre for Ageing and Neuroscience Stage II (29) (Cam-CAN: ages 18 to 88, *n* = 636). Leipzig showed a bimodal distribution of individuals older and younger than 50, which required statistical analyses of age as a discrete variable. Cam-CAN sampled more evenly across the life span, permitting additional statistical analyses of age as a continuous variable.

### Metabolic Neuroimaging Datasets.

To determine whether fuel affects brain network stability, we conducted resting-state scans on two independent cohorts of young healthy adults. Subjects were asked to keep their eyes open and let their minds wander while focusing on a white orienting cross on an otherwise black screen. To achieve the higher signal/noise required to analyze data at the single-participant level, participants were scanned using ultrahigh-field (7 T) fMRI at the Massachusetts General Hospital Athinoula A. Martinos Center for Biomedical Imaging, using acquisition parameters quantitatively optimized via dynamic phantom for detection sensitivity to resting-state networks (30). Immediately prior to and following each scan, blood glucose and ketone (d-β-hydroxybutyrate) levels were measured using Precision Xtra test strips (Abbott Laboratories) (Table 1). Exclusion criteria for all three studies included MR contraindications for ultrahigh-field imaging; diagnoses of psychiatric and/or neurological disorders; traumatic brain injuries; recreational drug usage, including severe alcohol use; and/or (for females) pregnancy. Participants were excluded if they were currently following or had recently followed (within past 6 mo) a low-carbohydrate or ketogenic diet. Detailed clinical and demographic characteristics for all individuals participating in the metabolic studies can be found in *SI Appendix*, Table S1. Studies were registered as a clinical trial on ClinicalTrials.gov (identifier NCT04106882) and approved by the institutional review boards of Massachusetts General Hospital (Partner’s Healthcare) and Stony Brook University; all participants provided informed consent. For access to relevant datasets (31) and code used to process and analyze the data, see Datasets S1–S4.

**Table 1.** Blood glucose and ketone measurements for MRS time course (*n* = 8) and fMRI bolus (*n* = 30) studies

	MRS time course study					
	Glucose bolus			Ketone ester bolus		
	PRE	POST 10 min	POST 80 min	PRE	POST 10 min	POST 80 min
Blood glucose, mg/dL (mmol/L)	91 ± 13 (5.1 ± 0.7)	95 ± 14 (5.3 ± 0.8)	89 ± 12 (4.9 ± 0.7)	89 ± 6 (4.9 ± 0.3)	82 ± 9 (4.6 ± 0.5)	67 ± 7 (3.7 ± 0.4)
Blood βHb, mmol/L	0.2 ± 0.1	0.2 ± 0.2	0.2 ± 0.1	0.1 ± 0.1	0.4 ± 0.4	3.6 ± 0.7
	Bolus study					
		Glucose bolus			Ketone ester bolus		
	PRE	POST 10 min	POST 50 min	PRE	POST 10 min	POST 50 min
Blood glucose, mg/dL (mmol/L)	95 ± 12 (5.3 ± 0.7)	101 ± 16 (5.6 ± 0.9)	90 ± 13 (5.0 ± 0.7)	92 ± 11 (5.1 ± 0.6)	91 ± 12 (5.1 ± 0.7)	74 ± 12 (4.1 ± 0.7)
Blood βHb, mmol/L	0.2 ± 0.1	0.2 ± 0.1	0.1 ± 0.1	0.1 ± 0.1	1.4 ± 1.2	3.5 ± 1.1

The Abbott Precision Xtra Glucose & Ketone Monitoring System was used for all fingerstick blood measurements. PRE = prebolus; POST = postbolus.

For the first experiment (diet) (*n* = 12, μ_age_ = 28 ± 7 y; 4 female), we scanned participants under three conditions: 1) standard diet: following their standard diet, without fasting; 2) fasting: following their standard diet, with an overnight (12 h) fast; and 3) ketogenic diet: following a ketogenic (high-fat, moderate-protein, low-carbohydrate [<50 g/d]) diet for 1 wk, by which point all participants were in ketosis (>0.6 mmol/L ketone blood concentration).

For the second experiment (bolus) (*n* = 30, μ_age_ = 29 ± 8 y; 18 female), we scanned an independent cohort of participants under three conditions: 1) fasting: following their standard diet, with an overnight fast; 2) glucose bolus: breaking the fast with a glucose drink (Glucose Tolerance Test Beverages, Fisher Scientific Inc.); and 3) d-βHb ketone ester bolus: breaking the fast with a ketone drink (d-β-hydroxybutyrate ketone ester; HVMN).

The d-βHb ketone ester was weight dosed for each participant at 395 mg/kg and calorically matched (μ_cal_ = 125 ± 19) between d-βHb ketone ester (μ_KETdose_ = 26.65 ± 3.97 g) and glucose (μ_GLUdose_ = 31.33 ± 4.57 g). Prior to neuroimaging, we acquired fasting plasma glucose and insulin measures for calculation of insulin resistance using HbA1c (μ_HbA1c_ = 5.14 ± 0.32% [min/max = 4.6 to 5.8%; insulin resistant > 5.6%]) and the Homeostatic Model Assessment of Insulin Resistance (HOMA-IR) (μ_HOMA-IR_ = 1.41 ± 0.59 [min/max = 0.41 to 2.87; insulin resistant > 2.0]). HOMA-IR was calculated as fasting insulin (μU/mL) × fasting glucose (mg/dL)/405 (32).

While ketone pharmacokinetics for human peripheral blood concentrations have been established (33), ketone pharmacokinetics for the human brain were unknown. Thus, to establish optimal timing for the bolus study, we first performed a magnetic resonance spectroscopy (MRS) study (*n* = 8; μ_age_ = 27 ± 5 y; 3 female) to determine the bolus time course in the brain. Using a within-participant time-locked design, as well as weight- and calorie-matched dosing as described below, we measured brain glucose and β-hydroxybutyrate at baseline, then every 5 min for 90 min after administering each bolus. As per *SI Appendix*, Fig. S2**,** MRS showed glucose and ketones reaching peak concentrations in the brain at ∼30 min postbolus. Of the two fuel types, glucose was confirmed to be shorter acting and more volatile compared to ketones (postpeak coefficient of variation was 2.1 ± 0.8 for glucose and 0.14 ± 0.03 for the d-βHb ketone ester; *P* = 0.04), which remained at their peak for at least 90 min postbolus. Thus, to ensure peak concentrations in the brain for both glucose and ketones, for the bolus experiment we acquired 10 min resting-state scans starting 30 min postbolus.

To check for potential interactions between diet and bolus conditions, as well as to test whether the d-βHb ketone ester could, in principle, counteract the effects of higher glycemic load, we conducted an additional investigation using one participant (case study: female, age 47, HbA1c = 5.8%). For the case study, the baseline condition consisted of a standard diet supplemented 30 min prior to the scan with a 75 g glucose bolus, a standardized challenge dose used clinically for the oral glucose tolerance test (34). In a time-locked within-subject design, the participant was scanned twice: on one day with a weight-dosed (395 mg/kg) 25 g d-βHb ketone ester bolus and on another day without it. Each of these two conditions was conducted at resting state and while performing spatial navigation and motor tasks, as described below.

### MRI Acquisition.

Both life span datasets were acquired at 3 T field strength; Leipzig had a time to repetition (TR) = 1,400 ms over 15 min and 30 s, while Cam-CAN had a TR = 1,970 ms over 8 min and 40 s [further details may be found in dataset documentation (28, 29)]. Given the focus on clinical applications, requiring single-participant-level resolution, all metabolic datasets were acquired at ultrahigh-field (7 T) field strength and included whole-brain BOLD (echoplanar imaging, EPI), field map, and T1-weighted structural (multi-echo magnetization prepared rapid gradient echo [MEMPRAGE]) images. BOLD images were acquired using a protocol quantitatively optimized, using a dynamic phantom (BrainDancer; ALA Scientific Instruments), for detection sensitivity to resting-state networks (30): Simultaneous multi-slice (SMS) slice acceleration factor = 5, *R* = 2 acceleration in the primary phase encoding direction (62 reference lines) and online generalized autocalibrating partially parallel acquisition (GRAPPA) image reconstruction, TR = 802 ms, echo time (TE) = 20 ms, flip angle = 33°, voxel size = 2 × 2 × 1.5 mm, slices = 85, and number of measurements = 2,320 in each of the prebolus and postbolus intervals, for a total acquisition time of 62 min. Field map images were acquired using the following parameters: TR = 723 ms, TE1 = 4.60 ms, TE2 = 5.62 ms, flip angle = 36°, voxel size = 1.7 × 1.7 × 1.5 mm, and slices = 89, for a total acquisition time of 3 min, 14 s. The whole-brain T1-weighted structural volumes were acquired with 1 mm isotropic voxel size and four echoes with the following protocol parameters: TE1 = 1.61 ms, TE2 = 3.47 ms, TE3 = 5.33 ms, TE4 = 7.19 ms, TR = 2,530 ms, and flip angle = 7°, with *R* = 2 acceleration in the primary phase encoding direction (32 reference lines) and online GRAPPA image reconstruction, for a total volume acquisition time of 6 min, 3 s.

### Spatial Navigation and Motor Tasks.

To assess whether effects extended beyond resting state to tasks that increased cognitive load and therefore brain metabolic demand, for the diet study and case study, participants additionally navigated virtual reality mazes using an MR-compatible joystick (Nata Technologies). We created these mazes using the Aldous–Broder algorithm in Daedalus (https://www.astrolog.org/labyrnth/daedalus.htm) and programmed them for a virtual reality scanner environment using Vizard (WorldViz). For the spatial navigation task, participants made use of spatial encoding and memory in finding their way from one end of the maze and back. For the motor task, participants simply followed a corridor and therefore navigated without making decisions.

### MRI Preprocessing.

Life span preprocessing was conducted in the FMRIB Software Library (FSL; https://fsl.fmrib.ox.ac.uk/fsl/fslwiki/): Anatomical images were skull stripped and coregistered to Montreal Neurological Institute (MNI) templates and mean functional images. Functional images were motion and field map corrected, brain extracted, and coregistered to MNI templates using transformations learned through the anatomical image. Metabolic preprocessing used Statistical Parametric Mapping 12 (SPM12; https://www.fil.ion.ucl.ac.uk/spm/software/spm12/) combined with an image processing workflow established with fMRIPrep (35). Anatomical images (MEMPRAGE) were normalized to MNI templates using unified segmentation and registration. Images of each individual participant were realigned to account for head movements and field map corrected for geometric distortions caused by the magnetic field inhomogeneity, followed by normalization to MNI space. Physiological confounds were removed using the Component Based Noise Correction Method (CompCor) (36). No spatial smoothing was applied to any of the datasets. For all datasets, voxelwise data were parceled into the Willard 499 functional region of interest (ROI) atlas, which further coarse grained data into 14 resting-state networks (37).

### fMRI Network Analyses.

To probe temporal dynamics and reorganization of communication across brain regions (interregional communication, typically described as brain networks, and those networks’ persistence over time, defined as network stability), ROI-level fMRI time series were binned into nonoverlapping time windows, or “snapshots,” of 24 s. From each window an all-to-all, signed, symmetric network of correlation strengths was extracted. We quantified the stability of brain networks in two complementary ways. To measure gross difference, we calculated total instability, defined as the (scalar) norm of difference in the correlation matrix for each pair of distinct snapshots of the brain network, where τ is the time duration (in units of 24 s) over which persistence was calculated (*SI Appendix*, Fig. S5). To identify which networks across the brain were most responsible for these effects, we calculated the least absolute shrinkage and selection operator (LASSO) regression on the feature set of instabilities calculated from resting-state networks and structural parcellations as defined by the Automated Anatomical Labeling atlas (AAL; http://www.gin.cnrs.fr/en/tools/aal-aal2/). These identified a data-driven construct, brain age, with the minimum number of coefficients. To measure large-scale functional reorganization, we calculated module instability for fMRI data acquired from the diet study (*n* = 12), indicating the extent to which nodes in a network module switched modules over time. Modules are defined as the nonoverlapping partition of all nodes in the network, such that intramodule connections are maximized relative to the intermodule connections. For each network matrix, modules were extracted using the Louvain parameter-free modularity-maximization algorithm (38). To obtain the (scalar) module instability, we averaged over all nodes: calculating the percentage of the node’s neighbors within the same module that failed to remain in the same module for the next network snapshot. Finally, to quantify general brain activity (39, 40), we calculated the whole-brain signal amplitude for low-frequency (0.01 to 0.1 Hz) fluctuations (ALFF).

## Results

### Brain Networks Destabilize with Age.

**Fig. 1.**
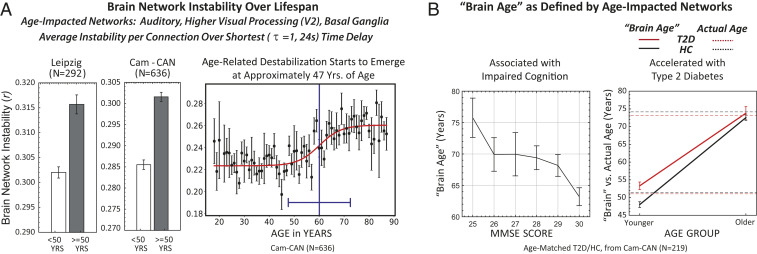
Brain networks destabilize with age, with the strongest impact in the auditory, higher visual processing (V2), and basal ganglia networks (total *n* = 928). (*A*) Leipzig Mind-Brain-Body open-source dataset, ages 20 to 85, binarized into younger (*n* = 214) vs. older (*n* = 78) participants (Mann–Whitney *U* = 0.28; *P* = 1.4 × 10^−8^) and Cam-CAN open-source dataset, ages 18 to 88, binarized into younger (*n* = 281) vs. older (*n* = 355) participants (Mann–Whitney *U* test = 0.27, *P* = 1.6 × 10^−22^). We fit network instability for the Cam-CAN dataset using a (logistic) sigmoidal function (nonlinear least squares with weights inversely proportional to the SD, reduced χ^2^ = 1.07). From this fit, we obtained the inflection point (switch point), which occurs at 60 ± 2 y, and the width accounting for 90% of the transition of 13 ± 6 y, resulting in an onset of degeneration at 47 y. A linear fit to the data resulted in a 30% larger reduced χ^2^ value, indicating that the data are more accurately fitted by a sigmoidal rather than linear fit. (*B*) Increasing brain age, defined by network stability, predicts progressively lower cognition (MMSE scores). Linear fit to brain age vs. MMSE score data finds a slope of –0.66 ± 0.27 (estimate ± SE), implying instability-derived brain age increases 0.66 y for every point decrease in MMSE score (*P* < 0.01, CI = [–1.18, –0.14]). For younger individuals, T2D accelerates brain aging compared to age-matched healthy controls. Mean actual ages for younger individuals with (51 ± 4 y, *n* = 14) and without (51 ± 5 y, *n* = 109) T2D were equivalent, while brain age for young diabetics was significantly increased over that of healthy controls (younger T2D vs. HC, Mann–Whitney *U* = 0.34, *P* = 0.0002). For older individuals, mean actual ages for T2D (73 ± 6 y, *n* = 14) and for HC (74 ± 6 y, *n* = 82) were equivalent to their respective brain ages (older T2D vs. HC, Mann–Whitney *U* test = 0.48, *P* = 0.87).

Across the life span measured by Cam-CAN, cognitive acuity declined with age, as measured by the standard clinical instrument used to assess dementia, the Mini-Mental State Examination (MMSE) (41) (*r* = –0.30, *P* = 3.63 × 10^−15^). Cam-CAN and Leipzig resting-state datasets show that increased age, in turn, was associated with destabilization of brain networks (Leipzig <50 y [*n* = 214] vs. ≥50 y [*n* = 78], Mann–Whitney *U* test = 0.28, *P* = 1.4 × 10^−8^; Cam-CAN <50 y [*n* = 281] vs. ≥50 y [*n* = 355], Mann–Whitney *U* test = 0.27, *P* = 1.6 × 10^−22^; Fig. 1 *A*, *Left*). This effect was driven primarily by the dynamics of three resting-state functional networks (37): auditory (superior temporal gyrus), higher visual processing (V2) and basal ganglia (thalamus, caudate, inferior frontal gyrus). LASSO regression with instability of the 12 resting-state networks as predictor variables and age as the predicted variable identified these three networks with high selectivity (*r* = 0.30, *P* = 7.11 × 10^−19^), assigning all other networks zero weight.

Age-associated degradation in network stability was sigmoidal (Fig. 1 *A*, *Right*; *n* = 636; sigmoid reduced χ^2^ = 1.07 vs. linear reduced χ^2^ = 1.39), with an inflection point of 60 ± 2 y, indicating the age at which network stability degraded most precipitously. The base of the sigmoid was 13 y earlier; thus, networks in our life span dataset started to destabilize at ∼47 y. Importantly, this suggests that the first latent markers for brain aging may be capable of neurobiological detection decades before cognitive symptoms become evident.

The three most dominantly affected networks were combined into a single variable, brain age, linearly composed of Cam-CAN-derived network stability values for the auditory (β_norm/raw_: 0.25/1.77), higher visual processing (β_norm/raw_: 0.35/2.51), and basal ganglia (β_norm/raw_: 0.16/1.19) networks. Brain age inversely correlated with cognitive acuity (Fig. 1 *B*, *Left*). Moreover, for younger individuals, T2D accelerated brain aging compared to age-matched healthy controls (Fig. 1 *B*, *Right*). Mean actual ages for younger individuals with (51 ± 4 y) and without (51 ± 5 y) T2D were equivalent, while brain age for young diabetics was significantly higher than that of healthy controls (younger T2D vs. HC, Mann–Whitney *U* test = 0.34, *P* = 0.0002). For older individuals, both mean actual ages for individuals with (73 ± 6 y) and without (74 ± 6 y) T2D and brain ages for the two groups (older T2D vs. HC, Mann–Whitney *U* test = 0.48, *P* = 0.87) were equivalent. Thus, younger individuals with T2D showed brain network destabilization (i.e., brain age) that, for nondiabetics, normally would be seen at an older age.

### Ketosis Stabilizes Brain Networks.

**Fig. 2.**
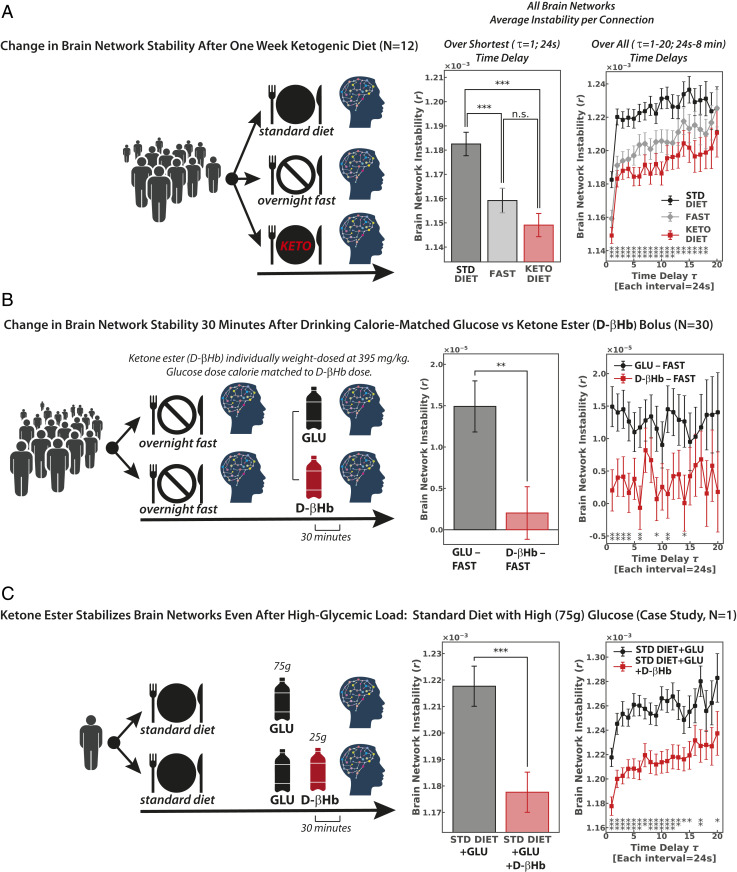
Brain networks destabilize with glucose and stabilize with ketones. (*A*) In the diet experiment, each participant was scanned three separate times, time locked to eliminate diurnal variability: while following a standard diet (STD), after overnight fasting, and after following a ketogenic diet for 1 wk (τ = 1, repeated-measures ANOVA LSD post hoc, standard vs. ketogenic diet: *t* = 5.4 *P* = 0.0000001). (*B*) To isolate fuel source as the variable of interest between the diets, we followed up with a bolus experiment. Each participant was scanned two separate times, again time locked to eliminate diurnal variability, with the d-βHb ketone ester individually weight dosed (395 mg/kg). Each individual’s glucose dose was then calorie matched to his or her d-βHb ketone ester dose. For each session we subtracted intrasession fasting values from each bolus value (τ = 1, paired *t* test, glucose bolus minus fasting vs. ketone ester bolus minus fasting: *t* = 2.9, *P* = 0.004). (*C*) The ketone ester’s stabilizing effects were observed even under high glycemic load; here we show network stability values for a single participant, following a standard diet that included a 75 g glucose challenge, with and without administration of the ketone ester (τ = 1, paired *t* test, high-glycemic standard diet with vs. without 25 g d-βHb ketone ester bolus: *t* = 4.12, *P* = 0.0001). Error bars for the case study (*n* = 1) reflect statistics calculated over up to 24 windows for τ = 1, 23 windows for τ = 2, etc. Equivalent effects for the same participant performing motor and spatial navigation tasks are shown in *SI Appendix*, Fig. S4. n.s., not statistically significant; *P ≤ 0.05; **P ≤ 0.01; ***P ≤ 0.0001.

Experimental modulation of fuel intake shows that brain networks are stabilized in healthy younger adults through ketosis both induced by a 1 wk change of diet (τ = 1, repeated-measures ANOVA least significant difference (LSD) post hoc, standard vs. ketogenic diet: *t* = 5.4, *P* = 0.0000001, *n* = 12; Fig. 2*A*) and as rapidly as 30 min following ingestion of exogenous d-βHb ketone ester (τ = 1, paired *t* test, glucose bolus–fasting vs. d-βHb ketone ester bolus–fasting, *t* = 2.9, *P* = 0.004, *n* = 30; Fig. 2*B*). Overall, both ketosis induced by a ketogenic diet and ketosis induced by drinking exogenous d-βHb ketone ester showed effects equivalent to those seen with fasting (τ = 1, repeated-measures ANOVA LSD post hoc, Diet_KET–FAST_: *P* = 0.75, *n* = 12; Bolus_KET–FAST_: *P* = 0.1, *n* = 30; Fig. 2 *A* and *B*), while a standard diet and glucose bolus consistently destabilized brain networks. As a measure, network stability showed robust test–retest reliability, with minimal intrasubject variation across the bolus study’s two fasting sessions spaced an average of 4 d (±2 d) apart (τ = 1, repeated-measures ANOVA LSD post hoc, fasting session 1 vs. fasting session 2, *P* = 0.28, *n* = 30; *SI Appendix*, Fig. S3).

Clearly visible even at the single-participant level, our case study showed the d-βHb ketone ester has brain network stabilizing effects even under high glycemic load (τ = 1, paired *t* test, standard diet+75g glucose bolus with vs. without d-βHb ketone ester bolus, *t* = 4.12, *P* = 0.0001; Fig. 2*C* and *SI Appendix*, Fig. S4). Blood values for the case study are provided in *SI Appendix*, Fig. S2 and Table S2.

**Fig. 3.**
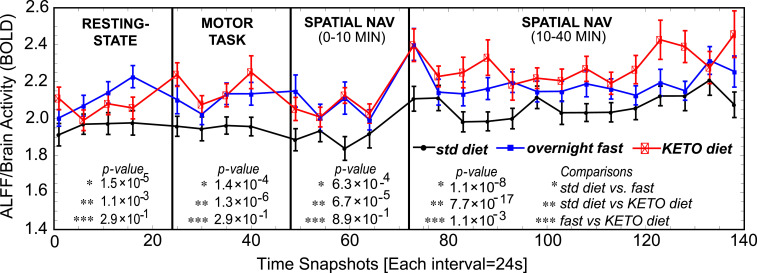
ALFF, a general measure of brain activity, was increased for participants on the ketogenic diet compared to their standard (std) diets (*n* = 12). This remained true for resting state, as well as during motor and spatial navigation tasks. Resting state and motor tasks were of 10 min duration. Spatial navigation shows the first 10 min (for comparison with other tasks) and then an additional 30 min, for 40 min total. This was done to assess fatigue effects over longer periods of time. Comparing symmetry over time between shifts from lower- to higher-activity states versus shifts from higher- to lower-activity states, both the ketogenic and fasting conditions showed a mean of zero bias (one-sample *t* test ketogenic diet: *t* = –0.22, *P* = 0.83; overnight fast: *t* = 0.26, *P* = 0.80), whereas the standard diet condition showed the brain switching from high- to lower-activity states (standard diet: *t* = –3.29, *P* = 0.007).

Further analyses showed network instability occurs from large-scale reorganization of network modules (network switching; *SI Appendix*, Figs. S5*A* and S6 *A* and *B*), rather than changing of connection strengths while preserving modules (network dimming or flickering; *SI Appendix*, Figs. S5*B* and S6 *C* and *D*). The fMRI signal’s ALFF, a general measure of brain activity, was consistently higher—across both rest and task conditions—for the participants following a ketogenic diet or fasting compared to following their standard diets (resting state: *P* = 1.1 × 10^−3^; motor task: *P* = 1.3 × 10^−6^; spatial navigation [early: 0 to 10 min]: *P* = 6.7 × 10^−5^; spatial navigation [late: 10 to 40 min]: *P* = 7.7 × 10^−17^, *n* = 12; Fig. 3). Across datasets, network switching became increasingly prominent with reduction of ALFF (diet: *r* = –0.39, *P* = 0.00003; Leipzig: *r* = –0.33, *P* = 2.63 × 10^−7^; Cam-CAN: *r* = –0.25, *P* = 4.15 × 10^−13^). Characterizing each network with respect to its total ALFF-derived activity for all nodes, we then compared symmetry for each “switch”: between transitions from lower- to higher-activity networks versus transitions from higher- to lower-activity networks. Both the ketogenic and fasting conditions showed mean zero bias (one-sample *t* test, keto diet: *t* = –0.22, *P* = 0.83; fast: *t* = 0.26, *P* = 0.80), whereas the standard diet condition biased the brain toward switching from higher- to lower-activity states (standard diet: *t* = –3.29, *P* = 0.007). Thus, network switching may reflect the brain’s inability to sustain the cost of more active, metabolically taxing, networks, thereby defaulting to metabolically “cheaper” (42) alternatives.

## Discussion

Our data provide evidence that, starting at around the age of 47 y, the stability of brain networks begins to degrade with age, with the most dramatic changes occurring around the age of 60 y. Since glucose hypometabolism remains one of the hallmark clinical features of dementia and its prodrome (43), we hypothesized that the network destabilization seen with aging might reflect the earliest stages of latent metabolic stress. Thus, we tested whether diets with different energetic yield might modulate network stability even in a younger population expected to be decades prior to any overt symptoms of age-based cognitive impairment. While glucose is normally considered to be the brain’s default fuel, β-hydroxybutyrate metabolism increases by 27% the Gibbs free energy change for ATP compared to glucose (23, 24). Consistent with that advantage, our results showed that even in younger (<50 y) adults, dietary ketosis increased overall brain activity and stabilized functional networks.

We first chose to manipulate diet in order to assess real-world clinical implications of food choices on the brain. However, change of diet within an ecologically realistic environment is a complex variable and therefore cannot dissociate whether the observed changes result from what is being taken away (carbohydrates) versus what is being added (fat) or even whether the changes might reflect different caloric intake (e.g., due to differences in satiety) for the two conditions. We thus followed up with a second study in which all participants followed their standard diets, fasted overnight, were scanned in a fasted state, and were then scanned again 30 min after drinking an individually weight-dosed and calorie-matched bolus: glucose on one day and d-βHb ketone ester on the other, counterbalanced for order. We found that the stabilizing effects seen with dietary ketosis were replicated with administration of exogenous ketones, which suggests that effects observed with modulating diet were specific to metabolism of glucose versus ketone bodies rather than more holistic changes seen between diets.

It should be noted that one difficulty in isolating the impact of each fuel type is the frequently observed (but potentially clinically beneficial in its own right) side effect of exogenous ketones in lowering glucose levels. This reflects a previously reported bias between the fuels: Ketone bodies, whenever present, are immediately utilized by the brain regardless of need, whereas glucose is only taken up by cells via GLUT transporters as required (15, 44). Thus, in the (inherently physiologically unnatural) state in which exogenous ketones are administered concomitantly with glucose, ketone bodies saturate cells, and the cerebral metabolic rate of glucose is down-regulated (44). However, ketone bodies would stabilize networks by lowering glucose levels only if glucose levels were already abnormally elevated, either due to insulin resistance or in response to a physiological perturbative bolus. The fact that network stabilizing effects were observed even in noninsulin resistant individuals—tested in a stable state of dietary glycolysis—suggests that those effects were consequent to ketosis rather than correcting a pathological state of hyperglycemia.

We next considered whether any systematic physiological effects of ketosis, such as diuresis (and therefore lowered blood pressure) or reduced cellular need for oxygen, might confound our fMRI results. However, if so, BOLD signal would have decreased in the ketogenic condition (45). The fact that ALFF and network stability increased during that condition suggests that the observed neurobiological effects did not result from global changes in hydration or oxygen. On the other hand, since ketone bodies have been shown to increase blood flow in the heart (46) and brain (47), an increase in cerebral blood flow would be consistent with increased BOLD, and therefore ALFF, but not with the network behavior we observed. Experiments combining arterial spin labeling and fMRI show increased cerebral blood flow is associated with increased fMRI connectivity (48), a modulation of connection strength. However, the observed, network instability reflects a qualitatively different behavior, in which networks transition between distinct topological configurations. We believe this behavior is more consistent with mechanisms of synaptic transmission, as suggested by previous animal experiments (19). Establishing potential mechanisms by which energy availability, at the cellular level, affects “rerouting” of neural signals will be an important future direction for multimodal and translational research.

For both diet and bolus experiments, d-βHb ketone ester and fasting conditions produced equivalent effects in stabilizing brain networks. Glycogen, when stored in the liver and skeletal muscle, typically sustains glycolysis for fasts of up to ∼30 h. However, the brain primarily utilizes glycogen stored in glia, which ^13^C MRS has shown in humans to become depleted in ∼5 to 10 h (49). Thus, following the typical overnight fast of ∼10 to 12 h, it is likely that the brains of non-insulin-resistant participants had already transitioned to endogenous ketosis, even if it was not yet detectable with assays of peripheral ketosis measured by blood or urine. Overall, our neuroimaging results support the hypothesis that at least some of the beneficial neural effects reported with hypocaloric states, such as intermittent fasting, severe caloric restriction, and exercise, may result from the brain’s transition to ketone bodies as fuel (50). While, for healthy individuals, the benefits of endogenous ketosis may be naturally achieved in multiple ways (e.g., ketogenic diet, fasting, exercise), this may not be necessarily true for those with insulin resistance, as chronically elevated insulin levels associated with insulin resistance—present even during fasting (32)—physiologically inhibit glucagon and therefore ketogenesis (51). Thus, while we showed endogenous and exogenous ketones to be qualitatively similar in stabilizing brain networks in young healthy adults, for insulin-resistant individuals, exogenous ketones may provide a useful adjunct in achieving the neurobiological benefits seen with endogenous ketosis, a further area for future study.

Finally, our focus on acute effects of modulating fuel source controlled for the role of several potential mechanisms associated with differences seen in large-scale epidemiological studies comparing diets. For example, insulin resistance has been suggested to indirectly facilitate vascular dementia, as hyperglycemia increases inflammation (52) and blocks nitric oxide (53), thereby effectively narrowing brain vasculature while also increasing blood viscosity (54). With respect to Alzheimer’s disease, recent results (55) have identified an insulin-degrading enzyme as playing a critical role in removing both excess insulin and amyloid β-protein from the brain. Since insulin and the protein compete with one another for the same enzyme, one consequence of the sustained high insulin levels associated with insulin resistance is depletion of the enzyme and therefore accumulated deposition of β-amyloid plaque. In addition, ketones have been shown to reduce inflammation and production of reactive oxygen species, as well as to up-regulate mitochondria in the brain. While all of these may have significant cumulative and synergistic effects in the months or years that precede cognitive impairment, it is striking how quickly the brain responded to a single week of dietary change or 30 min following a single dose of d-βHb. This rapid response effectively ruled out indirect inflammatory, antioxidant, tau/amyloid, and/or adaptive mitochondrial mechanisms of action, allowing us to isolate a more straightforward role of diet on metabolism. While further experiments will be needed to elucidate the mechanism at a microscopic scale and to explore its impact on the aging brain over longer time periods, the near-immediate changes in network stability, clearly visible even at the scale of the single participant, are encouraging, as they suggest that dietary interventions can have marked and measurable neurobiological effects on timescales relevant to clinical intervention.

## Supplementary Material

Supplementary File

## Change History

July 27, 2021: The data deposition footnote has been updated.
