# Supplementary material for: Diet modulates brain network stability, a biomarker for brain aging, in young adults
Source: Proc Natl Acad Sci U S A. 2020 Mar 3;117(11):6170–7. doi: 10.1073/pnas.1913042117 (PMC7084077; doi:10.1073/pnas.1913042117)
Supplement: Supplementary File [file pnas.1913042117.sapp.pdf]

PNAS
www.pnas.org

Supplementary Information for:

Diet modulates brain network stability, a biomarker for brain aging, in young adults.

Lilianne R. Mujica-Parodi, Ph.D.[1,2,3,4†], Anar Amgalan, M.S.[2,3†], Syed Fahad Sultan, B.S.[5], Botond Antal, B.S.[1], Xiaofei Sun, M.S.[5], Steven Skiena, Ph.D.[5], Andrew Lithen, B.S.[1], Noor Adra, B.S.[1], Eva-Maria Ratai, Ph.D.[4], Corey Weistuch, B.S.[2,6], Sindhuja Tirumalai Govindarajan, M.S.[1], Helmut H. Strey, Ph.D.[1,2], Ken A. Dill, Ph.D.[2], Steven M. Stufflebeam, M.D.[4], Richard L Veech, M.D. Ph.D.[7], Kieran Clarke, Ph.D.[8]

†Authors contributed equally to the submitted work.

1. Department of Biomedical Engineering, Stony Brook University, Stony Brook NY—USA 11794.
2. Laufer Center for Physical and Quantitative Biology, Stony Brook University, Stony Brook NY—USA 11794.
3. Department of Physics and Astronomy, Stony Brook University, Stony Brook NY—USA 11794.
4. A.A. Martinos Center for Biomedical Imaging, Massachusetts General Hospital and Harvard Medical School, Charlestown MA—USA 02129.
5. Department of Computer Science, Stony Brook University, Stony Brook NY—USA 11794.
6. Department of Applied Mathematics and Statistics, Stony Brook University, Stony Brook NY—USA 11794.
7. Laboratory of Metabolic Control, NIH/NIAAA, Rockville MD—USA 20852.
8. Department of Physiology, Anatomy, and Genetics, Oxford University, Oxford—UK. OX1 3PT.

Lilianne R. Mujica-Parodi, Ph.D.
Email: Lilianne.Strey@stonybrook.edu

This PDF file includes:

Supplementary text
Figures S1 to S6
Tables S1 to S2
Legends for Datasets S1 to S4
SI References

www.pnas.org/cgi/doi/10.1073/pnas.1913042117

Further Information Regarding Participants. Clinical and demographic characteristics of participants of the fMRI Diet and Bolus Studies, as well as the MRS Time-Course Study, are provided in **Table S1**. Blood results for the Case Study are provided in **Table S2.**

Establishing the Time-Course of Exogenous Ketones (D-β-Hydroxybutyrate). While the field has already established the time-course of exogenous D-βHb ketone ester in the bloodstream, the time-course for the fuel's passage via the blood-brain barrier was unknown. Thus, we first used magnetic resonance spectroscopy (MRS) to establish the time-course of the D-βHb ketone ester bolus, to ensure that we scanned only after it had reached its peak in the brain. We measured brain glucose and βHb using ^{1}H-MRS at baseline and for 90 minutes post-bolus on separate days in a within-subject design. MRS measurements were taken approximately every 5 minutes. Human participants (N=8) were scanned in a fasting state on a Siemens Trio 3T scanner using a 32Ch head coil. Participants were scanned for ten minutes to establish a glucose and βHb baseline and were then removed and asked to drink a bolus of D-βHb ketone ester (individually weight-dosed to 395 mg/kg) or glucose (dosed to same number of calories as D-βHb ketone ester). Single voxel spectroscopy sequences were collected from the BA24/32 region, corresponding to the *anterior cingulate cortex* (ACC), during resting state. We chose this region of interest (**Fig. S1**) because of its implication in insulin resistance(1) and normal aging(2, 3), and because its bilateral positioning removes the limitation of spectroscopy collection from both hemispheres, increasing time-resolution for the study. Long echo time (TE = 270 ms) PRESS sequences were used to measure the βHb time-course, while short echo time (TE = 30 ms) PRESS sequences were used for the glucose time-course. Manual and GRE shimming were used to optimize field homogeneity within the ROI. Time-courses for brain βHb and glucose were measured for 85 minutes following ingestion. Eddy-Current correction (ECC) and water suppression were performed using LCModel, and a basis set specific βHb or was developed and used to analyze the ketone-body data. As a standard, we collected Creatine (Cr)/ Phosphocreatine (PCr) values, which are relatively stable, and used their sum as an internal reference when calculating metabolite ratios. Timepoints 5 and 10 min were interpolated for all participants. As shown in **Fig. S2**, both average βHb and glucose levels peak around 30 minutes in the brain following the corresponding supplements. Of the two fuel types, glucose was confirmed to be shorter-acting and more volatile as compared to ketone bodies (post-peak coefficient of variation was 2.1 +/–0.8 for glucose and 0.14 +/–0.03 for the D-βHb ketone ester; p=0.04), which remained at their peak for at least 90 minutes post-bolus.

Test-Retest Reliability of the Instability Measure. During data collection for the *Bolus* experiment, we controlled for potential intra-subject variability across sessions by counterbalancing for order with respect to the glucose versus D-βHb ketone ester conditions. In addition, we presented our results in **Fig. 2B, Main Text** using baseline-correcting instability values for each session, in order normalize any residual intra-subject variability across sessions. In fact, participants' values across sessions showed high test-retest reliability, with the effects of the experimental manipulation far exceeding variance due to scans. We were able to assess this directly by using the "baseline" fasting scans acquired on both days, spaced an average of four days (+/– 2 days) apart. As shown by **Fig. S3** differences in brain network instability across the two days were not statistically significant (*paired t-test*; p=0.27, N=30), while pairwise comparisons for experimental conditions, using non-baseline-corrected methods equivalent to that used in the *Diet* experiment (**Fig. 2A, Main Text**), showed robust effects ($p<0.01$).

Case Study: Ketone Ester Stabilizes Brain Networks During Cognitive Load. In **Fig. 2C, Main Text** we illustrate detection sensitivity of the metabolic manipulation even at the single participant level, and showed that resting-state brain networks continue to be stabilized by a D-

βHb ketone ester bolus (individually weight-dosed for this participant at 25g) even when following a standard diet with high-glycemic load (induced with 75g glucose challenge, as per the standard oral glucose tolerance test for Type 2 diabetes). Here, we show (**Fig. S4**) that this effect holds not only during resting-state but during cognitive load as well. Participant (female, age 47, HbA1c=5.8%) navigated virtual reality mazes using an MR-compatible joystick (Nata Technologies; Coquitlam BC Canada). We created these mazes using the Aldous-Broder algorithm, in Daedalus (https://www.astrolog.org/labyrnth/daedalus.htm), and programmed them for a virtual reality scanner environment using Vizard (WorldViz, Santa Barbara CA). For the *spatial navigation task*, participant made use of spatial encoding and memory in finding her way from one end of the maze and back. For the *motor task*, she followed a corridor, and therefore navigated without making decisions.

Network Stability: What does it mean for a network to be "unstable"? If we define synchronous activity across brain regions, commonly representing synaptic signaling or "communication" across those regions, as *networks*, then the *stability* of those networks is the degree to which they persist over time. That persistance is denoted as units of time delay τ. Our typical results show differences between conditions for networks persisting up to approximately τ=18 for the *Diet* experiment and τ=11 for the *Bolus* experiment. Since each time interval during which the network is measured is itself 24s, the effects last about 264-432 seconds, or 4-7 minutes. One direction for future research is whether network stability—the length of time during which the brain is able to hold networks on line—underlies sustained attention, such that, as the stability of task-specific functional networks falter, it affects that ability to stay "on task".

A network can be unstable (i.e., change) in many ways. For example, and as illustrated by **Fig. S5**, the same network may change its topology (*switching*) while keeping constant the strength of its connections. Or it may retain the same topology while modulating connection strength (*dimming* if connection strength is decreased or increased over time, *flickering* if connection strengths alternate between decreasing and increasing). We hypothesize that, neurobiologically, network switching may reflect the brain's attempt to conserve energy by rerouting to paths with lower metabolic cost(4).

To measure large-scale functional reorganization, we calculated *module instability*, defined as the portion of nodes in a network module that switched modules between consecutive network snapshots. For each network matrix, "modules" (a non-overlapping partition of nodes in the network that are maximally intra-connected rather than inter-connected) were extracted using the Louvain parameter-free modularity-maximization algorithm(5). To obtain the (scalar) module instability, we averaged over all nodes: calculating the percentage of the node's neighbors within the same module that failed to remain in the same module for the next network snapshot.

As shown in **Fig. S6**, instability was characterized by switching between network modules (specifically, from higher-to-lower activity networks), rather than changing the connection strength (flickering or dimming) within the same network module over time.

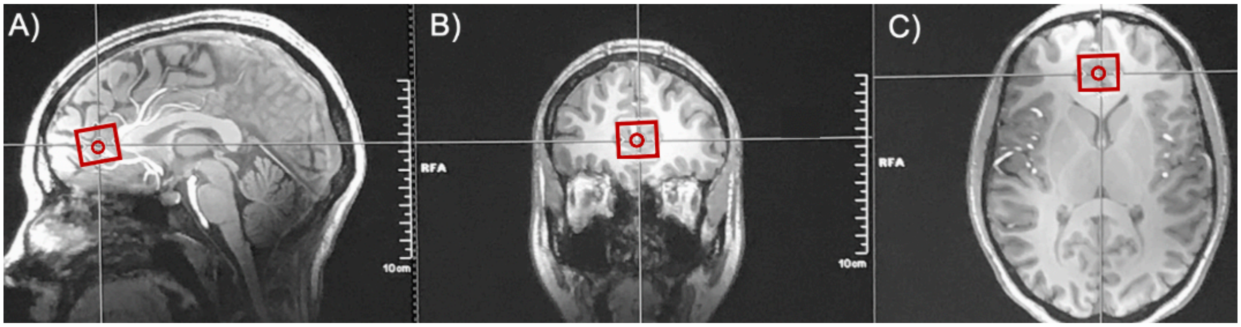

Figure S1: The region of interest for single voxel spectroscopy was obtained from the frontal lobe. The voxel (red box) was positioned in front of the corpus collosum and was centered on the anterior cingulate gyrus in the **A)** sagittal and **C)** axial planes, and on the lateral ventricle in the **B)** coronal plane.

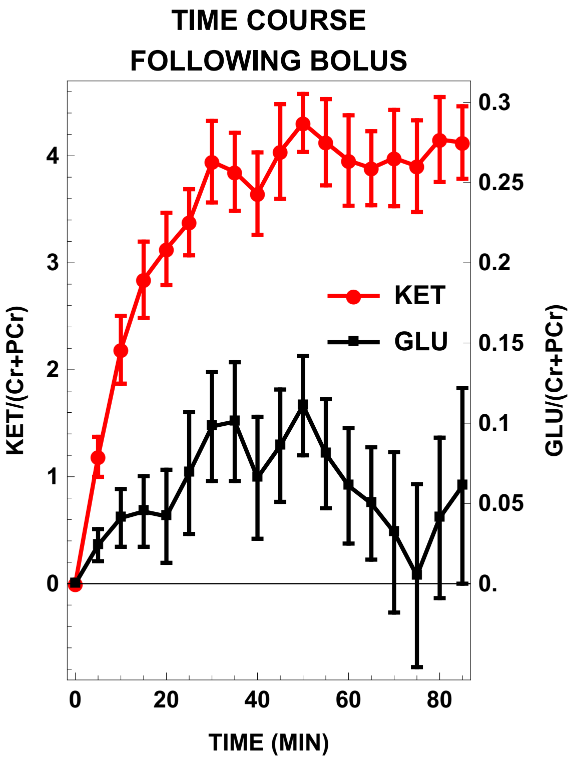

Figure S2: Brain glucose and D-βHb pharmacokinetic timecourse, as measured with ^{1}H Magnetic Resonance Spectroscopy. Average β-hydroxybutyrate (βHb/Cr+PCr) and glucose levels (Glc/Cr+PCr) in the brain (voxel centered on anterior cingulate gyrus) peak around 30 minutes following a ketone or glucose bolus and continue at peak for at least 90 minutes post-bolus. The increased volatility of glucose (as compared to ketones) in the brain is clearly visible and suggests that scans comparing the two fuels should occur between 30-40 minutes post-bolus, as per our study design.

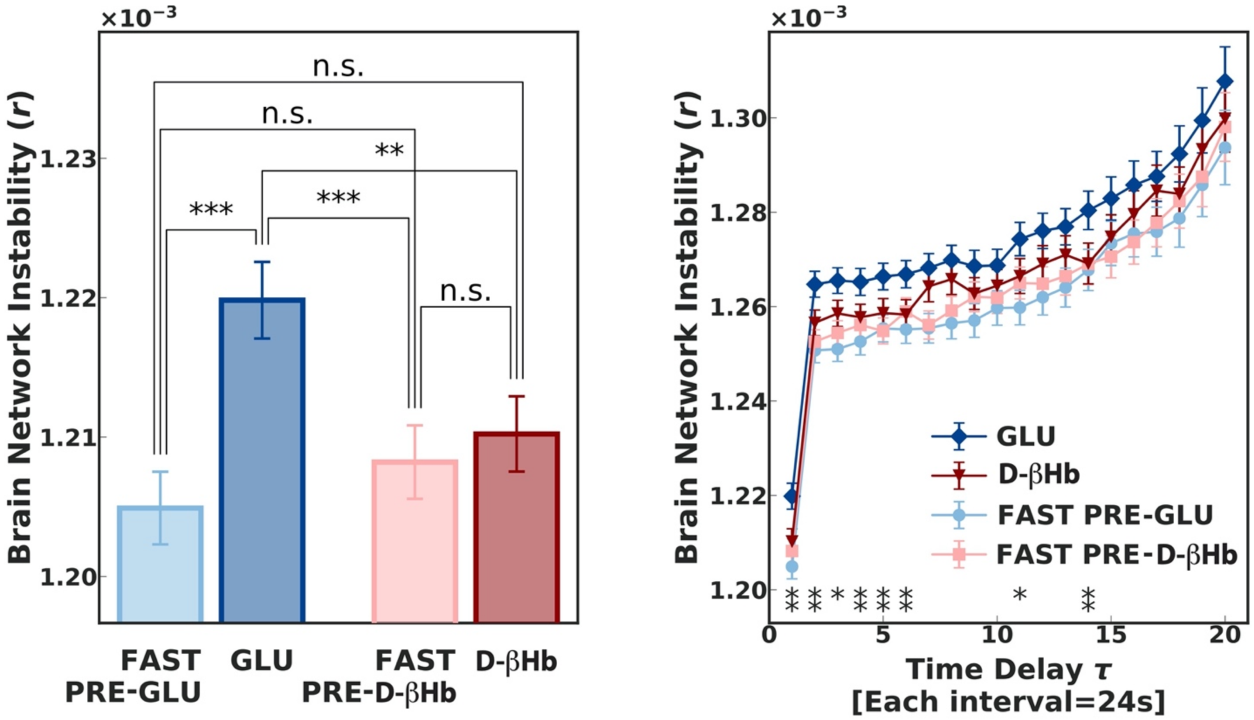

Figure S3: Baseline-corrected bolus results do not result from between-session variability in baseline. Using a within-subjects design (*Bolus* experiment, N=30), baseline (fasting) conditions on both days yielded equivalent network stability values. Non-baseline-corrected differences following administration of glucose vs. D-βHb ketone ester were statistically significant ($p \leq 0.01$). The glucose condition was significantly different from both (baseline) fasting conditions ($p \leq 0.001$). The fasting and D-βHb ketone ester conditions were equivalent.

Comparisons are for non-baseline corrected glucose versus D-βHb ketone ester conditions.
**p≤0.05; **p≤0.01; ***p≤0.001, n.s.=not statistically significant.*

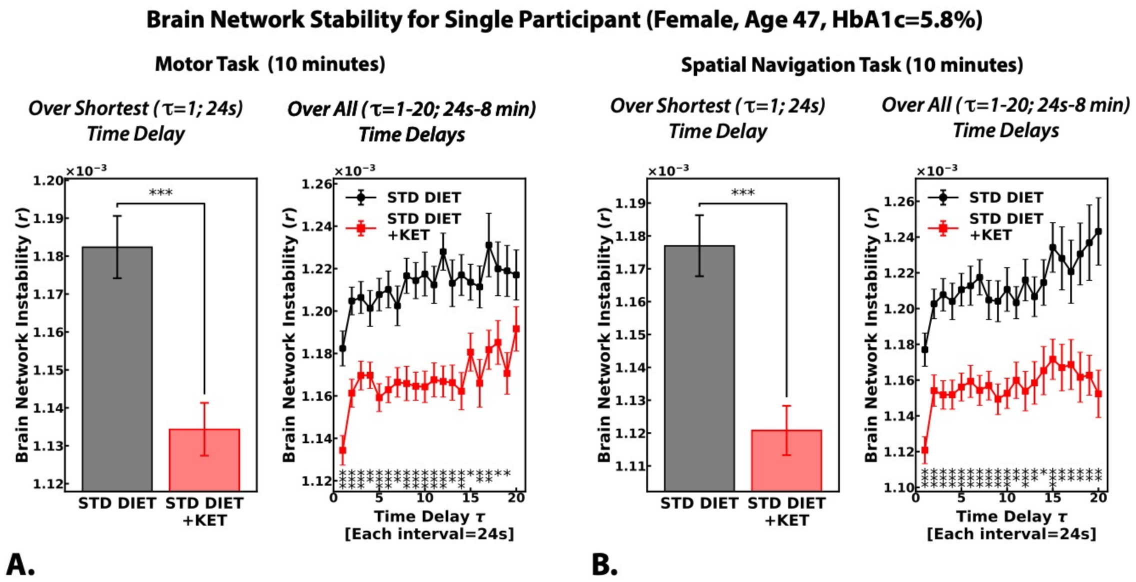

Figure S4: Single participant results for A) motor and B) spatial navigation tasks show that ketone ester shows comparable effects to this individual's resting state results (Main Text Fig. 2C), in stabilizing networks under cognitive load. Here, participant was tested under two conditions, following a standard diet with high glycemic load (75g glucose challenge) with and without administration of 25g D-βHb ketone ester bolus, with each 10-minute condition scanned three times consecutively. Equivalent effects were seen with boluses when calorie-matched (**Main Text, Fig. 2B**; N=30), which suggests the stabilization was not due to difference in calories between conditions.

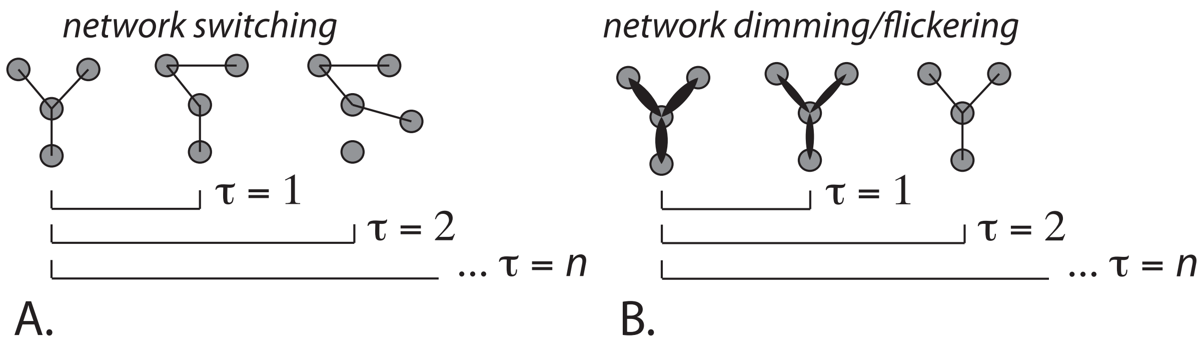

Figure S5: "Network stability" is defined as the degree to which edges persist over time-delay τ (where each τ is calculated in units of 24s). Thus τ =1 represents stability over 24s, τ=2 represents stability over 48s, etc. "Modules" are defined as a non-overlapping partition of all nodes in the network, such that the total strength of intra-module connections are maximized relative to the inter-module connections. To better understand what it means for brain networks to be unstable, we consider two possibilities: **A)** Network "switching," in which boundaries of the modules the network consists of are redrawn, thereby producing a new topology. **B)** Network "dimming/flickering," in which the network's partition into modules is preserved, while connection strengths are modulated.

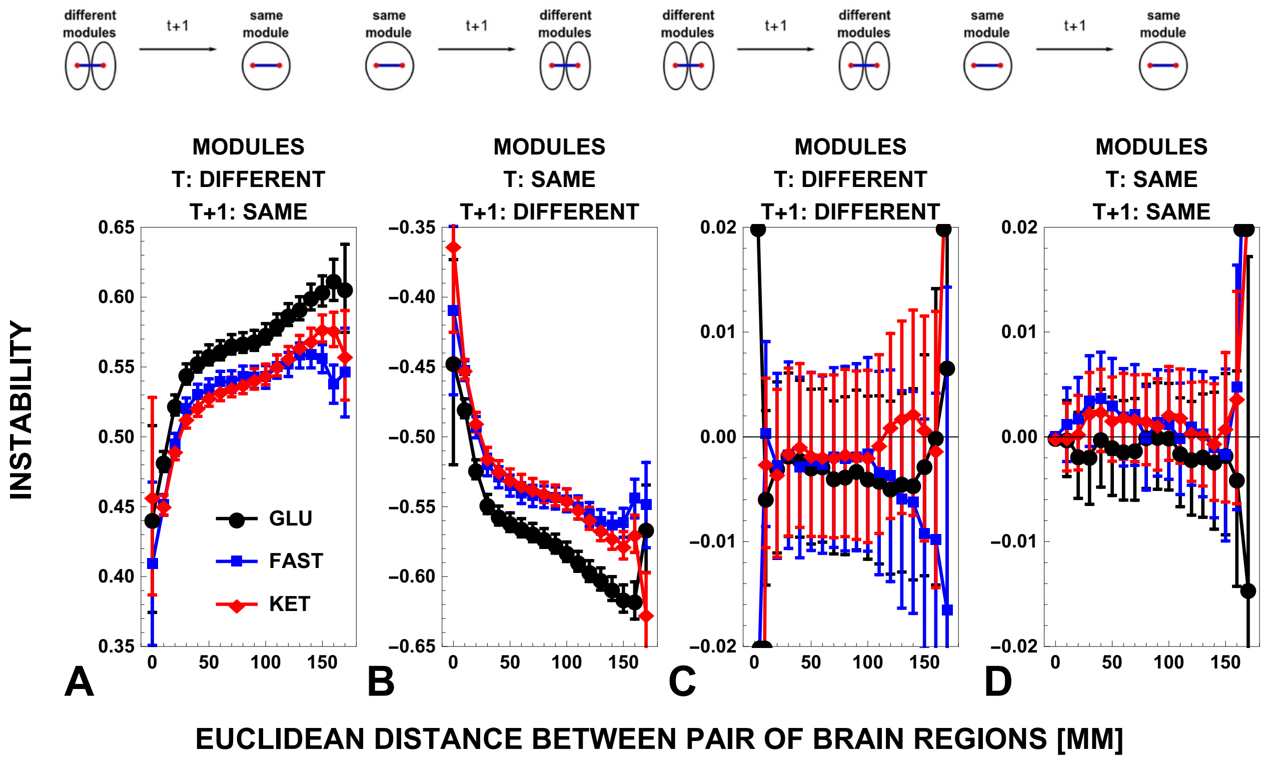

Figure S6. Instability of networks is driven largely by reorganization of brain's modular structure, and therefore network topology. Network connections **A)** between nodes that join into same module and **B)** between nodes that split into different modules show smaller amplitude change under ketosis and fasting condition. **A-B)** Both types of connections involved in module membership change undergo larger changes than **C-D)** connections between nodes whose module memberships do not undergo changes (compare scaling of vertical axes) and depend on Euclidean distance between nodes monotonically.

Clinical & Demographic Characteristics of the Study Participants

Measures		Dietary Study	Bolus Study	MRS Timecourse Study
Sex		N=12 (4 Female)	N=30 (18 Female)	N=8 (3 Female)
Age (years)	Average*	28 +/- 7	29 +/- 8	27 +/- 6
	Median	27	26	24
	Range	21-43	20-48	22-35
Ethnicity	White	11	15	3
	African-American	1	3	1
	Asian	0	7	2
	Hispanic	0	3	2
	Other	0	2	0
Body Mass Index (kg/m2)	Average	24.5 +/- 2.4	23.81 +/- 2.50	24.06 +/- 2.28
	Median	24.4	23.25	24.84
	Range	19.8-29.0	19.20-29.76	20.56-26.88
Body Fat (%)	Average		26.3 +/- 8.5	27.4 +/- 9.8
	Median		26.4	23.2
	Range		8.8-38.5	11.6-38.4
HbA1c (%)	Average		5.1 +/- 0.3	5.2 +/- 0.3
	Median		5.1	5.2
	Range		4.6-5.8	4.7-5.8
HOMA-IR	Average		1.4 +/- 0.6	1.4 +/- 0.8
	Median		1.27	1.11
	Range		0.41-2.87	0.83-2.87
Fasting Insulin (Plasma) (µIU/mL)	Average		6.8 +/- 2.6	6.6 +/- 3.2
	Median		6.4	5.7
	Range		2-13.5	4-13.5
Fasting Glucose (Plasma) (mg/dL)	Average		83 +/- 8 (4.6 +/- 0.4)	84 +/- 10 (4.7 +/- 0.6)
(mmol/L)	Median		84 (4.7)	86 (4.8)
	Range		64-105 (3.6-5.8)	68-105 (3.8-5.8)
Creatinine (mg/dL)	Average		0.87 +/- 0.16	0.99 +/- 0.20
	Median		0.88	0.98
	Range		0.6-1.32	0.6-1.32
eGFR (mL/min/1.73m2)	Average		106 +/- 14	92 +/- 21
	Median		108	107
	Range		67-127	67-116
Sodium (mmol/L)	Average		139 +/- 2	140 +/- 1
	Median		139	140
	Range		135-142	137-142
Potassium (mmol/L)	Average		4.1 +/- 0.4	4.0 +/- 0.2
	Median		4.0	4.1
	Range		3.6-4.9	3.6
Chloride (mmol/L)	Average		104 +/- 2	101 +/- 2
	Median		104	101
	Range		100-106	99-103
Carbon Dioxide (mmol/L)	Average		25 +/- 2	26 +/- 2
	Median		25	26
	Range		20-28	23-28
Calcium (mg/dL)	Average		9.3 +/- 0.4	9.4 +/- 0.4
	Median		9.3	9.5
	Range		8.6-9.8	8.8-9.8

** All Averages +/- SD*

Note: Health & Physical, Basic Metabolic Panel, and Oral Glucose Tolerance Test were conducted on all subjects during Bolus & Timecourse studies, within 30 days of MRI scan sessions for each participant (except the case study)

Table S1: Clinical and Demographic Characteristics of the Study Participants

CASE STUDY: Female, Age 47, HbA1c=5.8%								
	STD Diet + 75g GLU Bolus				STD Diet + 75g GLU Challenge + 25g D-βHb Bolus			
**Blood Glucose (mg/dL, mmol/L)**	159, 8.83	206, 11.4	190, 10.5	179, 9.94	163, 9.05	70, 3.89	145, 8.05	117, 6.49
**Blood Ketone (mmol/L)**	0.1	0.1	0.1	0.1	0.1	0.7	0.7	0.6
	Baseline	***1 Hr***	***2 Hrs***	***3 Hrs***	***Baseline***	***1 Hr***	***2 Hrs***	***3 Hrs***
		Post Bolus				*Post Bolus*		

Note: The Abbott Precision Xtra Glucose & Ketone Monitoring System was used for all fingerstick blood measurements.
GLU Bolus=75g dextrose; KET Bolus=25g ketone ester.

Table S2: Blood Glucose and Ketone Values for the Case Study Participant

SI Datasets

All datasets located at Data Archive for the Brain Initiative (DABI). (https://dabi.loni.usc.edu/explore/project/42) in the **Protecting the Aging Brain** (**PAgB**), **Project 1926781** repository. Additional details (including links to code used in the processing and analyses of data) can be found at: http://www.lcneuro.org/software-and-instrumentation.

- **Dataset S1: S1.0_Timecourse_3T_MRS_N8**
  (3T MRS Time-Course Study, N=8; μ_{age}=27 +/–5 years; 3 female)

Using a within-participant time-locked design, as well as weight and calorie-matched dosing as per LCNeuro-PAG 3 (described below), we measured brain glucose and β-Hydroxybutyrate at baseline, and then every five minutes for 90 minutes after administering each bolus. All conditions conducted at resting-state.

- **Dataset S2: S2.0_Diet_7T_FMRI_N12**
  (7T fMRI Diet Study N=12, μ_{age}=28+/–7 years; 4 female)

We scanned participants under three conditions: (1) *Standard diet*: following their standard diet, without fasting; (2) *Fasting:* following their standard diet, with an overnight (12 hour) fast; and (3) *Ketogenic Diet:* following a ketogenic (high-fat, moderate-protein, low-carbohydrate (<50g/day)) diet for one week, by which point all participants were in ketosis (>0.6 mmol/L ketone blood concentration). All conditions conducted at resting-state.

- **Dataset S3: S3.0_Bolus_7T_FMRI_N30**
  (7T fMRI Bolus Study, N=30, μ_{age}=29+/–8 years; 18 female).

We scanned an independent cohort of participants under three conditions: (1) *Fasting:* following their standard diet, with an overnight fast; (2) *Glucose Bolus:* breaking the fast with a glucose drink (Glucose Tolerance Test Beverages, Fisher Scientific, Inc.; Hampton NH); and (3) *D-βHb Ketone Ester Bolus:* breaking the fast with a ketone drink (D-β-hydroxybutyrate ketone ester; HVMN, San Francisco CA). The D-βHb ketone ester was weight-dosed for each participant at 395mg/kg, and calorically matched (μ_{cal}=125+/–19) between D-βHb ketone ester ($\mu_{KETdose}$=26.65g+/–3.97g) and glucose ($\mu_{GLUdose}$=31.33g+/–4.57g). Prior to neuroimaging, we acquired fasting plasma glucose and insulin measures, for calculation of insulin resistance using HbA1c (μ_{HbA1c}=5.14%+/–0.32% [min/max=4.6-5.8%; *insulin resistant* >5.6%]) and HOMA-IR ($\mu_{HOMA\text{-}IR}$=1.41+/–0.59 [min/max=0.41-2.87; *insulin resistant* >2.0]). HOMA-IR was calculated as: fasting insulin (µU/mL) x fasting glucose (mg/dL) /405(31). All conditions conducted at resting-state.

- **Dataset S4: S4.0_Case_7T_FMRI_N1**
  (7T fMRI Case Study, N=1, female, age 47, HbA1c=5.8%).

For the case study, the baseline condition consisted of a standard diet supplemented 30 minutes prior to the scan with a 75g glucose bolus—a standardized challenge dose used clinically for the oral glucose tolerance test. In a time-locked within-subjects design, the participant was scanned twice: on one day with a weight-dosed (395mg/kg) 25g D-βHb ketone ester bolus and on another day without it. Each of these two conditions was conducted at resting-state and while performing spatial navigation and motor tasks.

References

1. Willette AA, *et al.* (2013) Insulin resistance, brain atrophy, and cognitive performance in late middle-aged adults. *Diabetes Care* 36(2):443-449.
2. Resnick SM, Pham DL, Kraut MA, Zonderman AB, & Davatzikos C (2003) Longitudinal magnetic resonance imaging studies of older adults: a shrinking brain. *J Neurosci* 23(8):3295-3301.
3. Pareek V, Rallabandi VS, & Roy PK (2018) A Correlational Study between Microstructural White Matter Properties and Macrostructural Gray Matter Volume Across Normal Ageing: Conjoint DTI and VBM Analysis. *Magn Reson Insights* 11:1178623X18799926.
4. Trevisiol A, *et al.* (2017) Monitoring ATP dynamics in electrically active white matter tracts. *Elife* 6.
5. Blondel VD, Guillaume JL, Lambiotte R, & Lefebvre E (2008) Fast unfolding of communities in large networks. *J Stat Mech-Theory E*.
